# A case of molecularly profiled extraneural medulloblastoma metastases in a child

**DOI:** 10.1186/s12881-018-0526-8

**Published:** 2018-01-17

**Authors:** Nahla Ali Mobark, Musa Al-Harbi, Othman Mosleh, Sandro Santagata, Matija Snuderl, Malak Abedalthagafi

**Affiliations:** 1 Oncology Department, Cancer Centre, King Fahad Medical City, P.O. Box 59046, Riyadh, 11525 Kingdom of Saudi Arabia; 2 Department of Pathology, Brigham and Women’s Hospital, Harvard Medical School, Harvard Institute of Medicine, HIM-921F, 77 Avenue Louis Pasteur, Boston, MA 02115 USA; 3 Department of Pathology, New York University, Langone Medical Center, 240 E 38th Street, 22nd Floor, New York, NY 10016 USA; 4 Research Centre and Pathology Department, Saudi Human Genome Lab, King Fahad Medical City and King Abdulaziz City for Science and Technology, P.O. Box 59046, Riyadh, 11525 Kingdom of Saudi Arabia

**Keywords:** Extraneural metastasis, Medulloblastoma, *MYCN*-amplified, NGS, Methylation

## Abstract

**Background:**

Extraneural metastases are relatively rare manifestations of medulloblastoma.

**Case presentation:**

We present the case of a young boy with group three *MYCN*-amplified medulloblastoma. He received multimodal chemotherapy consisting of gross total resection followed by postoperative craniospinal radiation and adjuvant chemotherapy. The patient developed extraneural metastases 4 months after the end of therapy. Literature review identifies the poor prognosis of *MYCN*-amplified medulloblastomas as well as extraneural metastases; we review the current limitations and future directions of medulloblastoma treatment options.

**Conclusion:**

To the best of our knowledge, this is the first molecularly characterized report of extraneural metastases of medulloblastoma in a child.

## Background

Historically, medulloblastoma is the most frequent brain tumor in children accounting for more than 20% of pediatric brain tumor cases. Medulloblastoma can metastasize outside the nervous system and by direct extension via leptomeningeal seeding. The most common sites of metastasis are bone and bone marrow, followed by lymph nodes and, to a lesser extent, liver, lung, and peritoneum. Although only 1–2% of medulloblastoma patients present with extraneural metastasis at the time of initial diagnosis, the cumulative incidence during follow-up reaches values of up to 5–10%. To date, 282 patients with extraneural metastases have been reported. Of these reported cases, 40.4% have occurred in children [1, 2].

## Case presentation

**Fig. 1:**
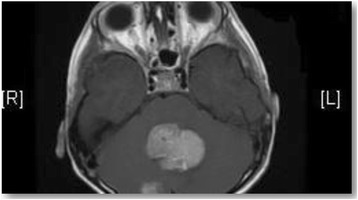
Brain MRI revealed a posterior fossa tumor with obstructive hydrocephalus

**Fig. 2:**
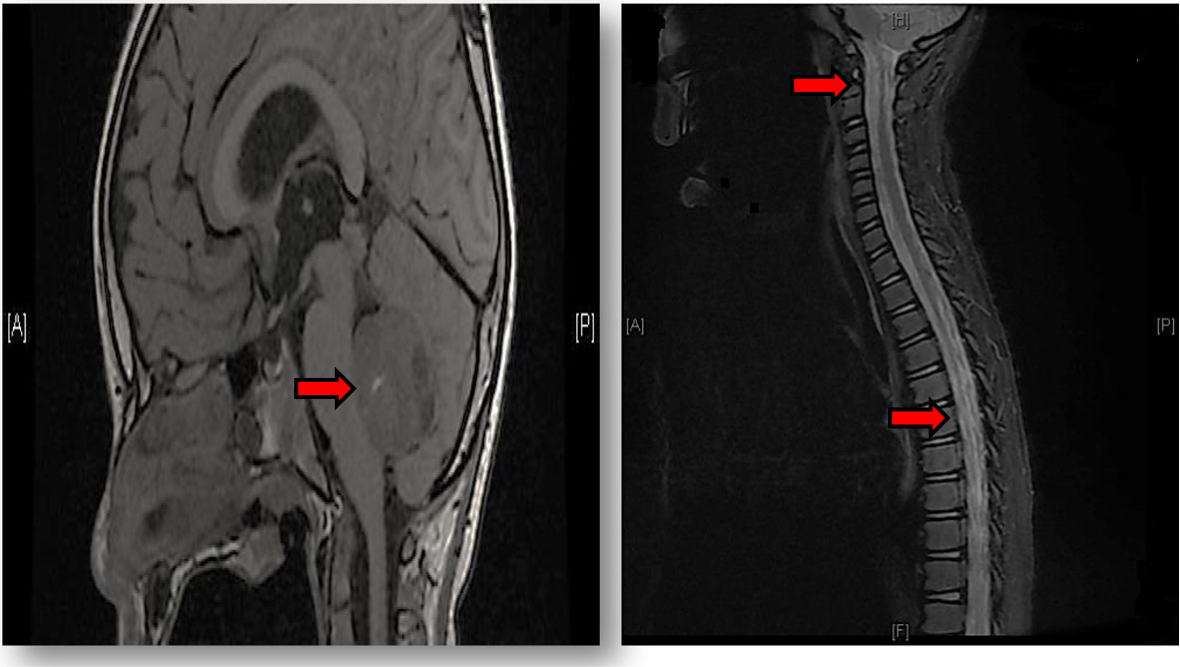
Brain and spine MRI showed a midline posterior fossa tumor suggestive of medulloblastoma with leptomeningeal enhancement concerning for intraspinal seeding (arrows)

A previously healthy 7-year-old boy was hospitalized in March 2015 with a 1-week history of progressively worsening symptoms of nausea, vomiting and decreased levels of consciousness. Upon admission, his examination was significant for diffuse hypotonia (though able to move all four limbs) and papilledema. A head CT scan revealed a posterior fossa tumor and obstructive hydrocephalus [Fig. 1]. An emergent EVD was placed to relieve the patient’s elevated intracranial pressure. Once clinically stable, brain and spine MRI was performed and showed midline posterior fossa tumors and leptomeningeal enhancement, suggestive of medulloblastoma with spine seeding, respectively [Fig. 2]. One week later, he had a craniotomy with gross total resection of the tumor on 26/3/2015. Histological diagnosis of the primary tumor was meduloblastoma, classic subtype.

The case was discussed at our institution’s multidisciplinary Neuro-Oncology tumor board. Plans were made to start multimodal therapy, as per the KFMC high-risk medulloblastoma protocol. This protocol consists of 6 weeks of craniospinal axis radiotherapy CSI 36 Gy and a boost to the posterior fossa to a dose of 55.8 Gy/ 31F concurrently with 2 cycles of oral Etoposide, beginning day 1 of radiation, oral Etoposide daily for 3 weeks every 4 weeks (i.e., 1 week off).

Repeat MRI was performed post radiation therapy and showed post-operative changes and interval improvement of leptomeningeal enhancement over the spinal cord. Adjuvant chemotherapy based on The COG ACNS0332 was started on week 11 and consisted of 3 cycles of Cisplatin and oral Etoposide (cycle A), and 3 cycles of cyclophosphamide and Vincristine (cycle B) for a total of 6 alternating cycles of A and then B, followed by 6 cycles of maintenance with oral Isotretinoin.

Our patient tolerated chemotherapy very well apart from one admission for febrile neutropenia following cycle B chemotherapy; he finished his chemotherapy in February 2016. Following treatment, the patient was in good condition, without symptoms suggestive of persisting disease or recurrence. End of therapy follow-up brain and spine magnetic resonance imaging (MRI) revealed no signs of recurrence.

However, 2 months after the completion of therapy, the patient began to complain of a hard and non-tender right mandibular swelling that gradually increased in size.

**Fig. 3:**
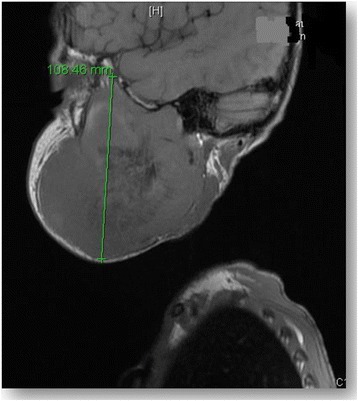
MRI of the mandibular area revealed a large necrotic and heterogeneous right mandibular soft tissue mass with extension into the infratemporal fossa (arrow)

Brain and spine MRI was performed and showed no signs of tumor recurrence in the surgical bed of the posterior fossa and no evidence of CSF seeding metastases along the spine. However, MRI of the mandibular area revealed a large necrotic and heterogeneous right mandibular soft tissue mass with extension into the infratemporal fossa [Fig. 3].

The patient was re hospitalized in June 2016 for mandibular mass incisional biopsy. Histological examination of the biopsy revealed small, round, blue tumor cells, consistent with metastasis of medulloblastoma. Immunohistochemistry was performed using commercially available antibodies according to standard protocols for paraffin-embedded formalin-fixed tissue. The slides were incubated with antibodies against GFAP, INI1, synaptophysin and P53. Histopathology confirmed the diagnosis of medulloblastoma with classic morphology. No anaplasia or excessive nodularity noted in both samples. Immunohistochemistry for the primary and metastatic tumors were positive diffusely for synaptophysin and scattered positive glial staining for GFAP. Staining for P53 was negative. Intact nuclear staining for INI1, which confirm the diagnosis of classic medulloblastoma and rule out atypical teratoid rhabdoid tumor diagnosis.

We used two methods in our molecular testing for the primary specimen to better classify the genomic subgrouping because the next generation sequencing method alone classified the tumor as either group 3 or 4. The metastatic specimen didn’t have enough materials for testing.

1. *Oncopanel assay*

Using a QIAamp DNA FFPE Tissue Kit (Qiagen, Valencia, CA), we obtained DNA from approximately 5–10 × 0.6 mm punches containing at least 80% tumor (1 mm diameter, Miltex, Plainsboro, NJ) from a formalin-fixed paraffin-embedded (FFPE) tissue block. The concentration of double-stranded DNA was quantified using a Quant-iT™ PicoGreen® dsDNA Assay Kit (Life Technologies, Carlsbad, CA). Next-generation targeted exon sequencing (*Oncopanel*) of 300 cancer-related genes was performed using Illumina-based methods as previously described [3].

**Fig. 4:**
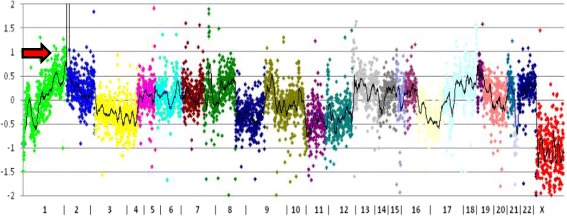
Plot of copy number variations by chromosomes that are color-coded. Sex chromosomes are excluded from the analysis. The vertical axis is the ratio of number of reads for a specimen to a panel of normal samples in log base 2 scales. A value of 0 denotes no difference from normal (diploid). When the sample contains 100% tumor cells, a value of − 1 is equal to 1 copy loss, and 0.58 is equal to 1 copy gain. The arrow shows the MYCN amplified region

*Oncopanel* was performed in a CLIA lab and considered a clinically validated genomics platform for variety of paediatric brain tumours including the medulloblastoma sub-classification. Two neuropathologists (MA and SS) reviewed the tumour specimen and estimated the number of neoplastic cells in the submitted sample (> 80% viable tumour cells). *Oncopanel assay showed* 9,564,899 unique, aligned, high-quality reads of this specimen, with a mean of 182 reads across all targeted exons and 98% of all exons having more than 30 reads. No notable somatic mutations were detected. Copy number analysis showed *MYCN* high amplification red arrow (Fig. 4); *ETV1* and *SOX9* high amplification (not shown); monosomies in chromosomes 3, 8, 10, 11, 16 and 21; and polysomy in chromosome 18. Breakmer analysis identified rearrangements involving (1) *MYCN* and a null area (Chr 2:16,086,243–2:18,295,773) and (2) *MYCN* and SMC6 (Chr 2:16,086,225–2:17,847,226). This molecular pattern suggests either group 3 or 4 medulloblastoma.

2. *Methylation assay*

Methylation data generated by the Illumina Infinium HumanMethylation 850 BeadChip array at the molecular pathology department in NYU. We used the previously described by the German Cancer Research Center (DKFZ), in-depth analysis of the DNA methylation [4, 5].

**Fig. 5:**
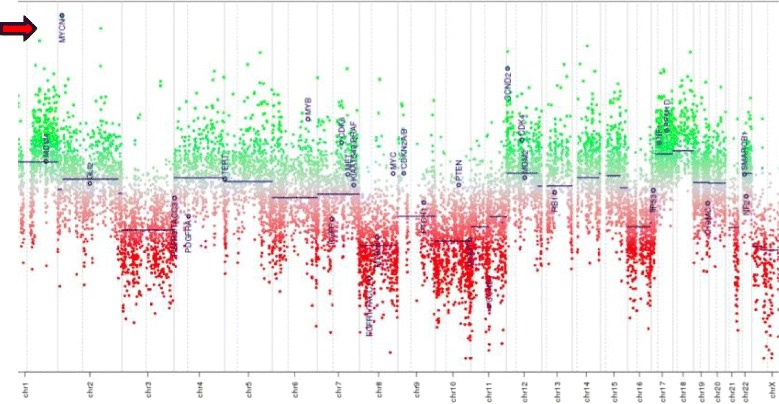
Methylation analysis for copy numbers analysis. Depiction of chromosome 1 to 22 (and X/Y if automatic prediction was successful). Gains/amplifications represent positive, losses negative deviations from the baseline. 29 brain tumor relevant gene regions are highlighted for easier assessment. Arrow highlights MYCN amplification

The brain tumor methylation classifier results (v11b2) reveled methylation class medulloblastoma, subclass group 3 with calibrated match score to the database of 0.99. The methylation class “medulloblastoma, subclass group 3” is comprised of tumors with the diagnosis medulloblastoma, genetically defined, group 3. Histologically most cases fall into the classical (like our case) and large cell/anaplastic groups. Copy number variation showed also *MYCN* amplification (arrow, Fig. 5).

The case was discussed in multidisciplinary Neuro-Oncology tumor board. Given his diagnosis of extraneural metastases, no further medical interventions were offered. Initially, his general condition was stable, and he continued palliative care at his home. After a couple of months, he was admitted again due to a rapid increase in the size of the mandibular mass. The mass showed extension into the oral cavity, with erosion and ulceration of the mucosa. The patient developed feeding difficulties, recurrent massive epistaxis and oral bleeding, he required frequent blood product transfusion, including recombinant factor VIIa, to control bleeding. Unfortunately, with further deterioration of his condition, he died 18 months after the initial diagnosis.

## Discussion and conclusions

Historically despite the heterogeneity of medulloblastoma, all patients receive the same therapy. The current standard of care for children with medulloblastoma involves maximal surgical resection and adjuvant therapy including both radiotherapy and chemotherapy. Approximately 80% of children with medulloblastoma are cured, but current treatment strategies leave medulloblastoma survivors with significant long-term morbidity and debilitating side effects. Frequently, cure of high-risk patients is difficult to achieve.

Medulloblastoma represent approximately 20% of all pediatric brain tumors and can be subdivided into 4 distinct molecular subgroups. These four distinct subgroups of medulloblastoma differ in demographics, somatic genetic events, & clinical outcomes. Current WHO 2016 classify four distinct subgroups of medulloblastoma with distinct demographics, genetics and clinical outcomes. These groups have been designated as Wnt-positive medulloblastoma, SHH-positive medulloblastoma, group 3 and group 4 [1].

Group 4 tumors comprise over 30% of all medulloblastoma and occur mainly in older children, though they can occur in adults and similar to group 3, more frequently occur in males. Group 4 medulloblastoma have a similar prognosis to SHH-positive tumors most groups 4 medulloblastoma have a classic histology. Group 3. Histologically most cases fall into the classical and large cell/anaplastic groups. Tumors are located in the cerebellum, typically in the vermis. Median age is 4 years (range 1 to 17). Group 3 medulloblastomas are more common in males than females. *MYC* amplification, aneuploidy, isochromosome 17q and *GFI1/1B* activation by enhancer hijacking are recurrent features, but a fraction lack an obvious driving genetic change [3–6].

Medulloblastoma metastases usually occur along the route of the cerebrospinal flow (i.e., the ventricular system and spinal cord). The extraneural metastases of primary brain tumors are relatively rare, accounting for 5–10% of all medulloblastoma cases.

Mechanisms of extraneural spread of medulloblastoma are still unclear. Craniotomy and surgical interventions mechanically disrupt the blood-brain barrier and allow migration of the tumor cells. Both hematogenic and lymphatic (involving cervical and retro auricular lymph nodes) spread of primary brain tumor cells have been suggested [7, 8]. Another frequently suggested mechanism for the extraneural spread of medulloblastoma is iatrogenic dissemination via ventriculoperitoneal shunts, which is likely to lead primarily to peritoneal metastases [8].

The majority of extraneural metastases of Medulloblastoma occur relatively early after initial diagnosis. While leptomeningeal and posterior fossa metastases are diagnosed in the first 5 years in 80–85% of the pediatric cases, nearly 80% of the extraneural metastases are discovered within the first 3 years after initial diagnosis [7, 8].

Concurrent central nervous system involvement, pulmonary or liver metastasis, early development of extraneural metastasis (< 18 months after initial diagnosis), and patient age of < 16 years at the time of extraneural metastasis diagnosis are all associated with poorer prognosis [8].

Recurrence patterns across medulloblastoma subgroups have been described, and it was found that medulloblastoma does not typically change subgroup at the time of recurrence, further highlighting the stability of the four-principle medulloblastoma subgroups [6, 8, 9]. Additionally, significant differences in the location and timing of recurrence across medulloblastoma subgroups were observed which have potential treatment ramifications. Specifically; *SHH*-positive tumors recur mostly in the local tumor bed, while group 3 and group 4 tumors recur mostly with extraneural metastases and do not relapse in the primary site, Group 4 tumors also have an increased time to death following recurrence.

These findings suggest that microscopic leptomeningeal metastases in group 3 and group 4 tumors not visible by neuroimaging or CSF examination are likely to be resistant to current therapy. Thus, additional local therapies targeting the primary site in the posterior fossa are unlikely to increase the cure rate for patients with group 3 or group 4 medulloblastoma, further reinforcing the importance of generating novel approaches to therapy in the metastatic compartment [8, 9].

The identification of molecular subgroups of medulloblastoma has potential to aid in understanding when it is possible to reduce standard chemotherapy doses and/or radiation therapies for children with favorable molecular medulloblastoma subgroups. Also intensify the therapy and /or use of maintenance-targeted therapies for high-risk medulloblastoma [10].

Recent study used highly integrative genomic analysis of medulloblastoma from 579 medulloblastoma samples, which led to identification of new subgroup-specific driver genes, epigenetic subtypes, and candidate targets for therapy [3].

The epigenetic defining group 3 and Group 4 MB subgroups are not as clearly demarcated as their WNT and SHH counterparts. This molecular heterogeneity could further help in predicting different clinical behavior in groups 3 and 4 and design better-targeted therapy. However, more clinical validation to this study is needed [3].

*C-MYC (MYC),* oncogene as well as its paralogs *MYCN* and *MYCL1*, has been shown to play essential roles in cycling progenitor cells born from proliferating zones during embryonic development. After birth, MYC plays important roles in the proliferation of all cell types*. MYC, MYCN*, and *MYCL1* amplifications have all been described in medulloblastomas and associated with poor prognosis [11–13].

Ongoing St. Jude MB protocol study (SJMB12) intensify therapy for stratum N3 High Risk patients with *MYC/MYCN* amplification who will receive high dose craniospinal radiation with boost to the primary tumor and standard chemotherapy (cisplatin, vincristine, cyclophosphamide) for 4 cycles intermixed with an additional 3 cycles of chemotherapy with pemetrexed and gemcitabine (https://clinicaltrials.gov/show/NCT01878617).

Novel agents are being tested as potential therapeutics for medulloblastoma. These agents include drugs that suppress expression of MYC isoforms by inhibiting bromodomain containing 4 (*BRD4*), a bromodomain. The preclinical evaluation of BET bromodomain inhibitors in MYCN-amplified medulloblastoma is also ongoing [14].

In summary, routine molecular profiling of medulloblastoma may give oncologists a more thorough understanding of tumors of unfavorable molecular subgroups that are at increased risk of extraneural metastases. This approach may help modify therapeutic approaches for children with unfavorable medulloblastoma molecular subgroups and have real implications for precision medicine in our oncology practice.

## Abbreviations

BRAD4: Bromodomain containing 4
EVD: External ventricular drain
FFPE: Formalin fixed embedded tissue
GFAP: Glial fibrillary acidic protein
INI-1: Integrase interactor 1
KFMC: King Fahad Medical City
MRI: Magnetic resonance imaging
MYC: V-Myc Avian Myelocytomatosis Viral Oncogene Homolog
NYU: New York University
